# Choice of methods can determine which behavioral determinates are identified for targeting in future behavior change interventions: Increasing antibiotic adherence in Pakistan

**DOI:** 10.1177/1359105320962267

**Published:** 2020-10-04

**Authors:** Umar Taj, Kelly Ann Schmidtke, Ivo Vlaev, Daniel Read

**Affiliations:** 1 The Business School, University of Warwick, UK; 2 The Medical School, University of Warwick, UK

**Keywords:** antibiotic, behavior change, health behavior, intervention, medication, Pakistan

## Abstract

When developing a behavioral intervention, formative research should be conducted to determine which behavioral barriers and facilitators to target. This is often done using qualitative interviews, but quantitative surveys may also be used. The current study examines the consequences of applying descriptive (rank order and *t*-tests) versus predictive (regression) quantitative analyses on intervention development, specifically for increasing antibiotic course completion. For demonstrative purposes, 1892 adults in Pakistan completed a cross-sectional survey that measures a comprehensive set of barriers/facilitators to their course completion. The descriptive and predictive analyses disagreed regarding which barriers/facilitators to prioritize. Reasons to prefer predictive analyses are discussed.

When developing complex behavioral interventions, formative research should be conducted to determine which behavioral influences (barriers and facilitators) to target (Campbell et al., 2000; Michie et al., 2014). Developing complex nationwide interventions can be daunting, because different individuals are affected by different behavioral factors to different degrees, yet it is rarely possible to customize interventions at the individual level. While different interventions can be rolled out in different locations, addressing all possible factors is often practically prohibitive and inefficient. Rather, we want to target those barriers and facilitators likely to generate the greatest population-level benefits. Theory can help, but the number of possible factors is often too great to be targeted in a single intervention (Francis et al., 2012). And, moreover, in no situation will all the potentially relevant factors be of equal causal relevance. This article contributes to our understanding of this problem by considering how different methods for selecting factors for targeting can lead to different conclusions. We do this in the context of the Theoretical Domains Framework (TDF), the leading theoretically driven framework for identifying key barriers and facilitators of behavior (Cane et al., 2012, 2015).

The TDF was developed by synthesizing 33 psychological theories of behavior change, thus helping interventionists take on a comprehensive and systematic theoretical approach, which promotes interventions’ effectiveness and sustainability (Atkins et al., 2017; Michie and Prestwich, 2010). The validated version of the TDF condenses 112 unique theoretical constructs about behavior change into 14 theoretically linked domains. These include: “Knowledge,” “Skills,” “Social/Professional role and identity,” “Beliefs about capabilities,” “Optimism,” “Beliefs about consequences,” “Reinforcement,” “Intentions,” “Goals,” “Memory attention and decision processes,” “Environmental context and resources,” “Social influences,” “Emotions,” and “Behavioral Regulation” (Cane et al., 2012, 2015; definitions in Supplemental Materials 1). In addition to being linked to theory, each domain is linked to one or more of the 93 empirically supported behavior change techniques best suited to leverage its underlying theoretical concepts, either directly or via an intervention function described by the Behavior Change Wheel (Michie et al., 2013; 2014). For example, the “Goals” domain is linked to the “commitment contracts” technique, which may increase people’s “implementation intentions.”

The TDF has been used to identify behavioral influences on medication adherence. Six examples are provided here. In Canada, a study examined adults’ adherence to medications that prevent myocardial infractions (Presseau et al., 2016). In England, a study examined adults’ adherence to nebulizer treatments for Cystic Fibrosis (Arden et al., 2019). In Northern Ireland, a study examined elderly adults’ adherence to multiple medications (Patton et al., 2018). In Scotland, a study examined homeless adults’ adherence to prescription medications (Paudyal et al., 2017). Studies looking specifically at antibiotic adherence have largely focused on health workers’ behaviors. In Ireland, a study examined health workers’ tendencies to prescribe antibiotics (Fleming et al., 2014), and in Australia, a study examined health workers’ use of delayed prescriptions (Sargent et al., 2017). The current study is the first to use the TDF to identify the behavioral influences on adults’ antibiotic course completion in a developing country: Pakistan.

When the current study was conducted antibiotic course completion was highlighted by the World Health Organization (2015) as a significant concern. Pakistan is one of the largest consumers of antibiotics, and 92% of the Pakistani population report not completing an antibiotic course (Atif et al., 2019; Klein et al., 2018). The current research team’s relationship with Gallup Pakistan made it possible to conduct a nationwide survey to examine how each TDF domain influences antibiotic course completion. More recent research may have led us to investigate antibiotic over-use (Llewelyn, 2017). However, the purpose of our paper is to use this behavior (i.e. antibiotic course completion) as an example to test methods, and not to advocate for a specific intervention for a specific behavior.

As is common in research using the TDF, most studies use qualitative interviews (Atkins et al., 2017). The six examples involving the TDF and medication adherence mention above all use qualitative interviews. The current research is focused on quantitative methods. Quantitative studies that apply the TDF to develop interventions have used descriptive analyses to describe the lowest and highest domain scores (e.g. Skoien et al., 2016) or to describe differences between types of participants at each domain (e.g. Stewart et al., 2018). While such analyses are informative, they may fall short of what is needed for effective intervention development. Rank order analyses may prove inadequate when the ranks do not reflect causal relationships with the intervention’s behavioral outcome, and comparisons across groups may prove inadequate when the same domains function as powerful influencers across groups.

As a demonstrative example, the current study compares the consequences of using descriptive and predictive quantitative analyses to determine which barriers/facilitators are targeted in an intervention to increase antibiotic course completion. We hypothesized that some domains would stand out in each analysis, and in particular sought to highlight differences that may influence intervention development.

## Methods

### Study design/setting

The current cross-sectional survey was conducted in May 2015 by Gallup Pakistan at participants’ homes in the Urdu language. The study was approved by the University of Warwick’s ethics committee (100/15-16). No identifiable information was made available to the research team, participation was voluntary, and consent was assumed for those who completed the survey. The study is reported according to the STROBE statement (Vandenbroucke et al., 2007).

### Participants

The research team planned to opportunistically recruit hundreds of participants, who indicated that they were at least 18 years old and had taken antibiotics. Participants were recruited across urban and rural locations. Locations were defined according to the Pakistan 2017 census, such that urban locations are “places with municipal corporation, town committee or cantonment” and rural locations are not (United Nations, Department of Economic and Social Affairs, Population Division, 2019: 94).

### Measurements

The survey measured participants’ self-reported antibiotic adherence and then their endorsement of statements about each TDF domain. Antibiotic adherence was defined for participants as *taking all the antibiotic medication provided without stopping in the middle*. Antibiotic adherence was assessed using Morisky et al.’s (1986) Medication Adherence Scale in which participants respond “yes” or “no” to each of four items, for example, “Do you ever forget to take your antibiotic medication?” The domains were assessed using 31 items informed by Huijg et al.’s (2014) validated template survey items (Supplemental Materials 1). Participants expressed their agreement with each item using a 10-point scale, from “strongly disagree” to “strongly agree.” Twenty-three items were positively worded such that higher scores indicated greater facilitators, and the remaining were reverse worded. Demographic information was also collected, including participants’ gender, location, age, and monthly income.

### Statistical methods

The analyses were conducted in SPSS v.26. Descriptive statistics were calculated to describe participant retention and demographics. Participants’ antibiotic adherence classifications were determined based on the number of “yes” responses given to the adherence scale: 3–4 = low, 1–2 = medium, and 0 = high. The retention, demographics, and adherence classifications of urban and rural participants were compared using two-sided Pearson’s Chi-square tests with a .05 alpha value.

Only participants who completed all the adherence and theoretical domain items were retained in the following analyses. Domain scores were produced by averaging the items within each domain to yield a mean score, along with its standard deviation; negatively worded items were reverse scored. The domain scores of urban and rural participants were compared using 14 independent-samples *t*-tests. Significance was assessed using a .05 alpha value, without applying Bonferroni’s correction due to the exploratory nature of formative research. Unequal variance was assumed where Levene’s test was significant, that is, less than .05 alpha. Next, two ordinal regressions were conducted to identify any domains, entered as covariates, that significantly predicted participants’ adherence classification (1 = low, 2 = medium, 3 = high), one for urban and one for rural locations. The significance of each predictor was assessed using a .05 alpha value.

### Data sharing statement

The current article includes the complete raw data-set collected including the participants’ data set, syntax file and log files for analysis. All of the data files are uploaded to the Figshare repository.

## Results

Out of the 1892 participants surveyed, 721 participants indicated having taken antibiotics (38.1%), and 549 (76.1%) of those participants completed all the items related to their adherence and the theoretical domains. Of these 549 participants (281 Female), 428 were from urban locations and 121 were from rural locations. The percentage of low adherers was similar across locations (41.4% urban vs 35.5% rural; *X2* (1, *N* = 549) = 1.33, *p* = .25). The lower sample sizes for rural locations entail that these findings are less reliable. Further details can be found in Supplemental Materials 2.

The bottom portion of the table in Supplemental Materials 2 provides the domain scores and standard deviations. Similar patterns appeared across locations. The two lowest scores across locations were for “Optimism” and “Rein-forcement” (*M*s range from 4.73 to 5.31), and the three highest scores across locations were for “Social/professional role and identity,” “Beliefs about consequences,” and “Knowledge” (*M*s range from 6.46 to 6.93). The t-tests revealed significant differences between locations at four domains, where positive t-values indicate higher score for Urban location: “Reinforcement” (*t*(547) = 2.72), *p* = 0.01, *d* = 0.29), “Memory Attention and Decision Processes” (*t*(547) = 3.54), *p* < 0.01, *d* = 0.37), “Skills” (*t*(246.6) = 2.72), *p* = 0.01, *d* = 0.26), and “Behavioral Regulation” (*t*(547) = −2.15), *p* = 0.02, *d* = 0.23). No other significant differences were located. Further details can be found in Supplemental Materials 3.

Next, the regression results are examined. For the urban location, the model was significant (*χ*^2^(14) = 57.52, *p* < 0.001), explaining 14.8% (Nagelkerke R2) of the variance. The test of parallel lines was not significant (*χ*^2^(14) = 10.26, *p* = 0.74). The mean variance inflation factor was 2.1 (range 1.2–3.2). Two domains predicted increased adherence: “Skills” with an odds ratio of 1.15 (95% CI: 1.03–1.28; Wald *χ*^2^(1) = 6.43, *p* = 0.01), and “Memory attention and decision processes” with an odds ratio of 1.27 (95% CI: 1.12 to 1.44; Wald *χ*^2^(1) = 13.28, *p* < 0.001). For the rural location, the model approached significance (*χ*^2^(14) = 22.75, *p* = 0.06), explaining 20.8% (Nagelkerke *R*^2^) of the variance. The test of parallel lines neared significance (*χ*^2^(14) = 23.35, *p* < 0.06). The mean variance inflation factor was 2.9 (range 1.2–7.1); Knowledge was the only domain with a variance inflation factor greater than 5. The same two domains predicted increased adherence: “Skills” with an odds ratio of 1.39 (95% CI: 1.06–1.82; Wald *χ*^2^(1) = 5.62, *p* = 0.02) and “Memory attention and decision processes” with an odds ratio of 1.47 (95% CI: 1.08–2.01; Wald *χ*^2^(1) = 5.84, *p* = 0.02). No other predictors were significant in either analysis. Further details can be found in Supplemental Materials 4.

## Discussion

The current study compared the consequences of descriptive and predictive analyses for intervention development. Each analysis identified different sets of barriers/facilitators to target. The rank order analysis identified the same five factors across locations: “Optimism,” “Reinforcement,” “Social/professional role and identity,” “Know-ledge,” and “Beliefs about consequences.” The *t*-tests suggested that “Reinforcement,” “Memory Attention and Decision Processes,” and “Skills” should be targeted for improvement in rural locations, and that “Behavioral Regulation” should be targeted in urban locations. The regression method identified two significantly influential domains across locations: “Skills” and “Memory attention and decision processes.” The current authors urge interventionists to use predictive analyses for quantitative studies. To develop effective interventions, interventionists need to do more than rank order or compare the domain scores: they need to understand the predictive relationships between each domain and the intended behavioral outcome or proxy measure (e.g. see Gibson Miller et al., 2020; Grady et al., 2018). The discussion now reviews previous research, some strengths and limitations of the study, and implications for future research and practice.

### Previous related research

The current study’s regressions identified two domains to prioritize. The qualitative studies mentioned in the introduction tended to identify more, and practical constraints often require that the number be reduced. Paudyal et al.’s (2017) interview findings suggested that 13 of the 14 domains influenced medication adherence, and then highlighted the six most mentioned domains as potential targets for future interventions. This choice seems odd, because the most mentioned domains may not be the most influential domains in terms of explaining variance of the target behavior. Patton et al.’s (2018) interview findings suggested that all 14 domains influenced adherence. Then they used group consensus to select a smaller number of domains they could feasibly target in their given context. It is unclear how much the interviews contributed to their ultimate decisions. While the quantitative method does not overcome all the uncertainty in making these practical choices, it does provide a more transparent process for informing them.

Arden et al.’s (2019) and Presseau et al.’s (2016) studies attempt to capture the predictive relationships between the domains and medication adherence. Arden et al. examined whether the number of times each domain was mentioned varied across participants’ adherence levels. As 12 of the 14 domains did, they were still left with a great number of domains to consider. Pressuau et al. first used qualitative interviews to identify the theoretical domains, and then used a quantitative survey to assess the predictive relationships between medication adherence and behavioral concepts described by a specific theory of behavior – the Health Action Process Approach (HAPA) – rather than the comprehensive TDF. Four HAPA concepts significantly predicted medication adherence, and the researchers used group consensus to map these concepts onto the “Social influences” and “Behavioral regulation” domains. This mapping process seems unnecessary given that the TDF domains can be quantified. A review of the HAPA is outside the scope of the current article (see Schwarzer, 2008). Here simply note that an advantage of using the TDF, as opposed to a specific theory, is that the TDF domains are the result of synthesizing of 33 theories of behavior change, and so cover more theoretically informed factors. In addition, the TDF domains are already linked to the empirically supported behavior change techniques most likely to influence each domain, as described by the Behavior Change Wheel (Michie et al., 2014).

### Implications for future research and practice

The current regression findings suggest that a nationwide intervention to increase antibiotic course completion in Pakistan should target “Skills” and “Memory attention and decision processes.” To leverage these domains, the Behavior Change Wheel recommends, for example, using techniques that restructure the environment (e.g. “prompts or cues” to take medication in the form of stickers placed on a bathroom mirror), or techniques that enable course completions (e.g. asking patients to write an “action plan” to take their medication). As the rank order analysis identified different domains, the Behavior Change Wheel recommends different techniques. For example, to leverage the “Optimism” domain, the Wheel recommends using persuasive techniques (e.g. providing “information about health consequences”). In addition, while the t-tests may lead interventionists to tailor techniques to each location’s unique needs, the regression analyses reveals that the same barriers/facilitators are influential across locations. Of course, if resources are more plentiful, less predictive domains could also be targeted, so long as they do not dilute each other’s effectiveness, which is a common problem in product/service development that Norman (2013) refers to as “creeping featurism.”

The current study encourages greater use of quantitative methods in formative research with the TDF. Beneficially, this move may “take some of the time burden away from using” the TDF (Phillips et al., 2015: 144). Within the current article, rank orders, *t*-tests, and regression analyses were considered, but other statistical analyses are available and may be appropriate depending on the research question, for example, structural equation modeling. The current recommendation is not strictly to use regressions and never to use rank orders or *t*-tests (nor is it to never use qualitative methods). Rather, we only seek to encourage the use of analyses that assess the predictive relationships between the domains and the intervention’s intended behavioral outcome when developing behavior change interventions.

### Strengths and limitations

A strength of the current study is the number of participants included in its analyses: 549. Of the four medication adherence studies described above involving the TDF, the largest number of participants was only 50 (Patton et al., 2018). While smaller numbers of participants may be sufficient to design interventions for homogenous groups, it seems unlikely that such small numbers would suffice to develop nationwide interventions. Another strength of the current study is its use of the TDF (Cane et al., 2012). As we discussed before, the TDF allows interventionists to consider a comprehensive set of theoretically informed behavioral domains that are linked to the empirically supported behavior change techniques best suited to leverage them (Michie et al., 2014).

Three limitations should be noted. One limitation is the current study’s focus on antibiotic under-use. Moving forward, formative research to understand antibiotic over-use in patients and prescribers should be conducted (Llewelyn, 2017). Second, participants’ self-reports are likely influenced by social desirability. The percentage of participants who indicated having taken antibiotic medication in the current study, 38%, seems lower than what one would expect given other findings. For example, nearly 80% of outpatient prescriptions in Pakistan contain at least one antimicrobial agent (Sarwar et al., 2018) and 33% of people admit to purchasing antibiotics without a prescription (Akhund et al., 2019). The third limitation involves there being three times more participants in urban than rural locations, and so the results at rural locations are less reliable. However, the lack of statistical precision should not stop interventionists from taking some action to improve public health. Rather, interventionists must consider this limitation as they determine what actions to take.

In conclusion, the current study examined the consequences of descriptive versus predictive quantitative analyses on which barriers/facilitators are selected for behavior change interventions. The particular domains identified across analyses differed, and so this choice can have profound consequence on intervention development. The current research team recommends using predictive quantitative analyses to understand each domain’s causal relationship with their intervention’s intended behavioral outcome.

## Research Data

Behaviour_Insigh_Data_EXCEL_For_JofHP9.28 – for Choice of methods can determine which behavioral determinates are identified for targeting in future behavior change interventions: Increasing antibiotic adherence in Pakistan Click here for additional data file. Behaviour_Insigh_Data_EXCEL_For_JofHP9.28 for Choice of methods can determine which behavioral determinates are identified for targeting in future behavior change interventions: Increasing antibiotic adherence in Pakistan by Umar Taj, Kelly Ann Schmidtke, Ivo Vlaev and Daniel Read in Journal of Health Psychology This article is distributed under the terms of the
Creative Commons Attribution 4.0 License ( https://creativecommons.org/licenses/by/4.0/)
which permits any use, reproduction and distribution of
the work without further permission provided the original
work is attributed as specified on the SAGE and Open
Access pages ( https://us.sagepub.com/en-us/nam/open-access-at-sage).

Behaviour_Insigh_Data_For_JofHP9.28 – for Choice of methods can determine which behavioral determinates are identified for targeting in future behavior change interventions: Increasing antibiotic adherence in Pakistan Click here for additional data file. Behaviour_Insigh_Data_For_JofHP9.28 for Choice of methods can determine which behavioral determinates are identified for targeting in future behavior change interventions: Increasing antibiotic adherence in Pakistan by Umar Taj, Kelly Ann Schmidtke, Ivo Vlaev and Daniel Read in Journal of Health Psychology This article is distributed under the terms of the
Creative Commons Attribution 4.0 License ( https://creativecommons.org/licenses/by/4.0/)
which permits any use, reproduction and distribution of
the work without further permission provided the original
work is attributed as specified on the SAGE and Open
Access pages ( https://us.sagepub.com/en-us/nam/open-access-at-sage).

Explanatory_Memo_Analysis_Guide_31-July-2020 – for Choice of methods can determine which behavioral determinates are identified for targeting in future behavior change interventions: Increasing antibiotic adherence in Pakistan Click here for additional data file. Explanatory_Memo_Analysis_Guide_31-July-2020 for Choice of methods can determine which behavioral determinates are identified for targeting in future behavior change interventions: Increasing antibiotic adherence in Pakistan by Umar Taj, Kelly Ann Schmidtke, Ivo Vlaev and Daniel Read in Journal of Health Psychology This article is distributed under the terms of the
Creative Commons Attribution 4.0 License ( https://creativecommons.org/licenses/by/4.0/)
which permits any use, reproduction and distribution of
the work without further permission provided the original
work is attributed as specified on the SAGE and Open
Access pages ( https://us.sagepub.com/en-us/nam/open-access-at-sage).

Log_File_Analysis_Guide_31-July-2020 – for Choice of methods can determine which behavioral determinates are identified for targeting in future behavior change interventions: Increasing antibiotic adherence in Pakistan Click here for additional data file. Log_File_Analysis_Guide_31-July-2020 for Choice of methods can determine which behavioral determinates are identified for targeting in future behavior change interventions: Increasing antibiotic adherence in Pakistan by Umar Taj, Kelly Ann Schmidtke, Ivo Vlaev and Daniel Read in Journal of Health Psychology This article is distributed under the terms of the
Creative Commons Attribution 4.0 License ( https://creativecommons.org/licenses/by/4.0/)
which permits any use, reproduction and distribution of
the work without further permission provided the original
work is attributed as specified on the SAGE and Open
Access pages ( https://us.sagepub.com/en-us/nam/open-access-at-sage).

Syntax_Analysis_Guide_31-July-2020 – for Choice of methods can determine which behavioral determinates are identified for targeting in future behavior change interventions: Increasing antibiotic adherence in Pakistan Click here for additional data file. Syntax_Analysis_Guide_31-July-2020 for Choice of methods can determine which behavioral determinates are identified for targeting in future behavior change interventions: Increasing antibiotic adherence in Pakistan by Umar Taj, Kelly Ann Schmidtke, Ivo Vlaev and Daniel Read in Journal of Health Psychology This article is distributed under the terms of the
Creative Commons Attribution 4.0 License ( https://creativecommons.org/licenses/by/4.0/)
which permits any use, reproduction and distribution of
the work without further permission provided the original
work is attributed as specified on the SAGE and Open
Access pages ( https://us.sagepub.com/en-us/nam/open-access-at-sage).

Supplementary_Materials – Supplemental material for Choice of methods can determine which behavioral determinates are identified for targeting in future behavior change interventions: Increasing antibiotic adherence in Pakistan Click here for additional data file. Supplemental material, Supplementary_Materials for Choice of methods can determine which behavioral determinates are identified for targeting in future behavior change interventions: Increasing antibiotic adherence in Pakistan by Umar Taj, Kelly Ann Schmidtke, Ivo Vlaev and Daniel Read in Journal of Health Psychology
