# Supplementary material for: Choice of methods can determine which behavioral determinates are identified for targeting in future behavior change interventions: Increasing antibiotic adherence in Pakistan
Source: J Health Psychol. 2020 Oct 4;27(4):1006–13. doi: 10.1177/1359105320962267 (PMC8855384; doi:10.1177/1359105320962267)
Supplement: Explanatory_Memo_Analysis_Guide_31-July-2020 – for Choice of methods can determine which behavioral determinates are identified for targeting in future behavior change interventions: Increasing antibiotic adherence in Pakistan [file Explanatory_Memo_Analysis_Guide_31-July-2020.docx]

**Explanatory Memo**

1. Relevant items in the data file have already been reverse coded.

2. SPSS files of the data have been made available.

3. The ordinal regressions were conducted the OMS and Plum procedure as described at Laerd Statistics.

Laerd Statistics (n.d.). Ordinal Regression using SPSS Statistics. https://statistics.laerd.com/spss-tutorials/ordinal-regression-using-spss-statistics.php#:~:text=Ordinal%20Regression%20using%20SPSS%20Statistics%20Introduction%20Ordinal%20logistic,dependent%20variable%20given%20one%20or%20more%20independent%20variables. (Located 27-July-2020).

4. An EXCEL file of the main SPSS data file has been made available.

| Column | Column Content | Column Code |
| --- | --- | --- |
| A | Number.Code | ID code to match across files |
| B | Blank.Before.Eligibility.items |  |
| C | Ever.Taken.Antibiotics.YES.NO | 1 = YES, 2 = NO, 99 = Don’t know |
| D | Completed.all.four.Medication.Adherence.items | 0 = NO, 1 = YES |
| E | Completed.all.31.TDF.items | 0 = NO, 1 = YES |
| F | Eligible | 0 = NO, 1 = YES |
| G | Blank.Before.Demographics | Blank for visual clarity |
| H | Gender | 1 = Male, 2 = Female |
| I | Location | 1 = Urban, 2 = Rural |
| J | Age Bracket | 1 = <30, 2 = 30 to 50, 3 = 50+, 99 = No response |
| K | Monthly Income Bracket | 1 = Up to 7000, 2 = 7001 to 1500, 5 = 15001 to 30000, 6 = More than 30000, 99 = no response |
| L | Blank.Before.Medication.Adherence.Low vs Not Low | Blank for visual clarity |
| M | MMAS_Low.vs.other.Adherers | 1 = Low adherer, 0 = Not low adherer |
| N | Blank.Before.TDF.Items | Blank for visual clarity |
| O | Knowledge_item 1 | 1 to 10, 99 = no response |
| P | Knowledge_item 2 | 1 to 10, 99 = no response |
| Q | Skills_item 1 | 1 to 10, 99 = no response |
| R | Social/ProfRole_item 1 | 1 to 10, 99 = no response |
| S | Beliefs about cap_item 1 | 1 to 10, 99 = no response |
| T | Beliefs about cap_item 2 | 1 to 10, 99 = no response |
| U | Beliefs about cap_item 3 | 1 to 10, 99 = no response |
| V | Optimism_item 1 | 1 to 10, 99 = no response |
| W | Optimism_item 2 | 1 to 10, 99 = no response |
| X | Beliefs about Consequences_item 1 | 1 to 10, 99 = no response |
| Y | Beliefs about Consequences_item 2 | 1 to 10, 99 = no response |
| Z | Reinforcement_item 1 | 1 to 10, 99 = no response |
| AA | Reinforcement_item 2 | 1 to 10, 99 = no response |
| AB | Intentions_item 1 | 1 to 10, 99 = no response |
| AC | Goals_item 1 | 1 to 10, 99 = no response |
| AD | Goals_item 2 | 1 to 10, 99 = no response |
| AD | Memory attention and decision processes_item 1 | 1 to 10, 99 = no response |
| AF | Memory attention and decision processes_item 2 | 1 to 10, 99 = no response |
| AG | Memory attention and decision processes_item 3 | 1 to 10, 99 = no response |
| AH | Environmental context and resources_item 1 | 1 to 10, 99 = no response |
| AI | Environmental context and resources_item 2 | 1 to 10, 99 = no response |
| AJ | Environmental context and resources_item 3 | 1 to 10, 99 = no response |
| AK | Social Influences_item 1 | 1 to 10, 99 = no response |
| AL | Social Influences_item 2 | 1 to 10, 99 = no response |
| AM | Social Influences_item 3 | 1 to 10, 99 = no response |
| AN | Emotions_item 1 | 1 to 10, 99 = no response |
| AO | Emotions_item 2 | 1 to 10, 99 = no response |
| AP | Emotions_item 3 | 1 to 10, 99 = no response |
| AQ | Emotions_item 4 | 1 to 10, 99 = no response |
| AR | Behavioural Regulations_item 1 | 1 to 10, 99 = no response |
| AS | Behavioural Regulations_item 2 | 1 to 10, 99 = no response |
| AT | Blank.Before.Medication.Adherence.Coded.for.Paper | Blank for visual clarity |
| AU | MMAS_Q1 | 0 = No, 1 = YES |
| AV | MMAS_Q2 | 0 = No, 1 = YES |
| AW | MMAS_Q3 | 0 = No, 1 = YES |
| AX | MMAS_Q4 | 0 = No, 1 = YES |
| AY | MMAS_Score | Sum of previous four columns: 3-4=low, 1-2=medium, and 0=high. |
| AZ | MMAS_Adherence_Category | 1 = High, 2 = Med, 3 = Low |
| BA | MMAS_Adherence_Category_For.Ordinal.Regression | 1 = Low, 2 = Med, 3 = High |
| BB | Blank.Before.TDF.Domains.for.Paper | Blank for visual clarity |
| BC | Knowledge | Mean of Knowledge Items |
| BD | Skills | Mean of Skills items |
| BE | Social.Professional.Role.and.Identity | Mean of Social/Prof Role items |
| BF | Beliefs.in.Capabilities | Mean of Beliefs in Cap items |
| BG | Optimism | Mean of Optimism items |
| BH | Beliefs.in.Consequences | Mean of Beliefs in Con items |
| BI | Reinforcement | Mean of Reinforcement items |
| BJ | Intentions | Mean of Intentions items |
| BK | Goals | Mean of Goals items |
| BL | Memory.Attention.and.Decision.Processes | Mean of Memory items |
| BM | Environmental.Contexts.and.Resources | Mean of Environment items |
| BN | Social.Influences | Mean of Social Influence items |
| BO | Emotions | Mean of Emotions items |
| BP | Behavioral.Regulation | Mean of Behavioural Regulation items |
