# Supplementary material for: Choice of methods can determine which behavioral determinates are identified for targeting in future behavior change interventions: Increasing antibiotic adherence in Pakistan
Source: J Health Psychol. 2020 Oct 4;27(4):1006–13. doi: 10.1177/1359105320962267 (PMC8855384; doi:10.1177/1359105320962267)
Supplement: Log_File_Analysis_Guide_31-July-2020 – for Choice of methods can determine which behavioral determinates are identified for targeting in future behavior change interventions: Increasing antibiotic adherence in Pakistan [file Log_File_Analysis_Guide_31-July-2020.docx]

**Log File of Statistical Outputs**

**(SPSS version 26)**

**Table of Contents.**

**Participant Characteristics, Rank Orders, and T-tests**

1. Frequencies = Total number of participants surveyed.

2. Frequencies = Number of participants indicating having taken antibiotics.

3. Frequencies = Number of participants completing all medication adherence and TDF items, i.e. ‘eligible’ participants.

4. Frequencies = Number of eligible participants identifying as female.

5. Frequencies = Number of eligible participants in each location (rural or urban).

6. Crosstabs = Chi Square test comparing percentage of low adherers at each location.

7. Descriptive = At each location, each domain’s mean scores and standard deviation scores.

8. T-tests = Comparing location domains scores at each domain.

**Ordinal Regressions**

9. Plum = Ordinal Regressions at Urban and Rural Locations

10. Regression = Conducted to assess multicollinarity of predictor domains.

11. Odds ratios = Picture of odds ratios outputs which was taken from a generated dataset: “Behaviour Insigh Data_Regression.PLUM_For JofHP9.28”

**Supplementary Materials 1. Table**

12. Frequencies = Total number of participants surveyed at each location.

13. Crosstabs = Chi square test comparing percentage of participants having taken antibiotics at each location.

14. Crosstabs = Chi square test comparing percentage of participants who completed all survey items (i.e. eligible) at each location.

15. Crosstabs = Chi square test comparing percentage of eligible participants who identified as female at each location.

16. Crosstabs = Chi square test comparing percentage of eligible participants across age groups at each location.

17. Crosstabs = Chi square test comparing percentage of eligible participants across adherence categories at each location.

18. Crosstabs = Chi square test comparing percentage of eligible participants across income categories at each location.

FREQUENCIES VARIABLES=Number.Code

/FORMAT=NOTABLE

/STATISTICS=SUM

/ORDER=ANALYSIS.

**1. Frequencies - Total Number of Participants Surveyed**

| **Statistics** | | |
| --- | --- | --- |
| Number.Code | | |
| N | Valid | 1892 |
|  | Missing | 0 |
| Sum | | 1790778.00 |

FREQUENCIES VARIABLES=nq6.Ever.Taken.Antibiotics.YES.NO

/STATISTICS=SUM

/ORDER=ANALYSIS.

**2. Frequencies - Number of Participants indicating having taken antibiotics**

| **Statistics** | | |
| --- | --- | --- |
| Please tell me have you ever taken the antibiotic medication? | | |
| N | Valid | 1888 |
|  | Missing | 4 |
| Sum | | 3055.00 |

| **Please tell me have you ever taken the antibiotic medication?** | | | | | |
| --- | --- | --- | --- | --- | --- |
|  | | Frequency | Percent | Valid Percent | Cumulative Percent |
| Valid | Yes | 721 | 38.1 | 38.2 | 38.2 |
|  | No | 1167 | 61.7 | 61.8 | 100.0 |
|  | Total | 1888 | 99.8 | 100.0 |  |
| Missing | DK/NR | 4 | .2 |  |  |
| Total | | 1892 | 100.0 |  |  |

USE ALL.

COMPUTE filter_$=(nq6.Ever.Taken.Antibiotics.YES.NO = 1).

VARIABLE LABELS filter_$ 'nq6.Ever.Taken.Antibiotics.YES.NO = 1 (FILTER)'.

VALUE LABELS filter_$ 0 'Not Selected' 1 'Selected'.

FORMATS filter_$ (f1.0).

FILTER BY filter_$.

EXECUTE.

FREQUENCIES VARIABLES=Eligible

/STATISTICS=SUM

/ORDER=ANALYSIS.

**3. Frequencies - Number of Participants completing all medication adherence and TDF items, i.e. Eligible participants**

| **Statistics** | | |
| --- | --- | --- |
| Eligible | | |
| N | Valid | 721 |
|  | Missing | 0 |
| Sum | | 549.00 |

| **Eligible** | | | | | |
| --- | --- | --- | --- | --- | --- |
|  | | Frequency | Percent | Valid Percent | Cumulative Percent |
| Valid | No-Not Eligible for analyses | 172 | 23.9 | 23.9 | 23.9 |
|  | Yes-Eligible for analyses | 549 | 76.1 | 76.1 | 100.0 |
|  | Total | 721 | 100.0 | 100.0 |  |

USE ALL.

COMPUTE filter_$=(Eligible = 1).

VARIABLE LABELS filter_$ 'Eligible = 1 (FILTER)'.

VALUE LABELS filter_$ 0 'Not Selected' 1 'Selected'.

FORMATS filter_$ (f1.0).

FILTER BY filter_$.

EXECUTE.

DATASET ACTIVATE DataSet1.

SAVE OUTFILE='\\WIMPLE\User54\b\bssmbf\Desktop\Behaviour Insigh Data_For JofHP9.28.sav'

/COMPRESSED.

DATASET ACTIVATE DataSet1.

SAVE OUTFILE='\\WIMPLE\User54\b\bssmbf\Desktop\Behaviour Insigh Data_For JofHP9.28.sav'

/COMPRESSED.

FREQUENCIES VARIABLES=sex

/STATISTICS=SUM

/ORDER=ANALYSIS.

**4. Frequencies - Number of Eligible Participants identifying as Female**

| **Statistics** | | |
| --- | --- | --- |
| Gender | | |
| N | Valid | 549 |
|  | Missing | 0 |
| Sum | | 830 |

| **Gender** | | | | | |
| --- | --- | --- | --- | --- | --- |
|  | | Frequency | Percent | Valid Percent | Cumulative Percent |
| Valid | Male | 268 | 48.8 | 48.8 | 48.8 |
|  | Female | 281 | 51.2 | 51.2 | 100.0 |
|  | Total | 549 | 100.0 | 100.0 |  |

FREQUENCIES VARIABLES=ur

/STATISTICS=SUM

/ORDER=ANALYSIS.

**5. Frequencies - Number of Eligible Participants in each Location**

| **Statistics** | | |
| --- | --- | --- |
| Location | | |
| N | Valid | 549 |
|  | Missing | 0 |
| Sum | | 670 |

| **Location** | | | | | |
| --- | --- | --- | --- | --- | --- |
|  | | Frequency | Percent | Valid Percent | Cumulative Percent |
| Valid | Urban | 428 | 78.0 | 78.0 | 78.0 |
|  | Rural | 121 | 22.0 | 22.0 | 100.0 |
|  | Total | 549 | 100.0 | 100.0 |  |

CROSSTABS

/TABLES=ur BY MMAS_Low.vs.other.Adherers

/FORMAT=AVALUE TABLES

/STATISTICS=CHISQ

/CELLS=COUNT

/COUNT ROUND CELL.

**6. Crosstabs - Chi Square test comparing percentage of low adherers at each location**

| **Case Processing Summary** | | | | | | | | | | | | |
| --- | --- | --- | --- | --- | --- | --- | --- | --- | --- | --- | --- | --- |
|  | | | Cases | | | | | | | | | |
|  |  |  | Valid | | | Missing | | | | Total | | |
|  |  |  | N | Percent | | N | | Percent | | N | | Percent |
| Location * MMAS_Low.vs.other.Adherers | | | 549 | 100.0% | | 0 | | 0.0% | | 549 | | 100.0% |
| **Location * MMAS_Low.vs.other.Adherers Crosstabulation** | | | | | | | | | | |  |  |
|  | | | | | MMAS_Low.vs.other.Adherers | | | | Total | |  |  |
|  |  |  |  |  | Not a low adherer | | Low Adherer | |  |  |  |  |
| Location | Urban | Count | | | 251 | | 177 | | 428 | |  |  |
|  |  | % within Location | | | 58.6% | | 41.4% | | 100.0% | |  |  |
|  | Rural | Count | | | 78 | | 43 | | 121 | |  |  |
|  |  | % within Location | | | 64.5% | | 35.5% | | 100.0% | |  |  |
| Total | | Count | | | 329 | | 220 | | 549 | |  |  |
|  |  | % within Location | | | 59.9% | | 40.1% | | 100.0% | |  |  |

| **Chi-Square Tests** | | | | | |
| --- | --- | --- | --- | --- | --- |
|  | Value | df | Asymptotic Significance (2-sided) | Exact Sig. (2-sided) | Exact Sig. (1-sided) |
| Pearson Chi-Square | 1.330^a^ | 1 | .249 |  |  |
| Continuity Correction^b^ | 1.098 | 1 | .295 |  |  |
| Likelihood Ratio | 1.343 | 1 | .246 |  |  |
| Fisher's Exact Test |  |  |  | .293 | .147 |
| Linear-by-Linear Association | 1.327 | 1 | .249 |  |  |
| N of Valid Cases | 549 |  |  |  |  |
| a. 0 cells (.0%) have expected count less than 5. The minimum expected count is 48.49. | | | | | |
| b. Computed only for a 2x2 table | | | | | |

SORT CASES BY ur.

SPLIT FILE LAYERED BY ur.

DESCRIPTIVES VARIABLES=Knowledge Skills Social.Professional.Role.and.Identity

Beliefs.in.Capabilities Optimism Beliefs.in.Consequences Reinforcement Intentions Goals

Memory.Attention.and.Decision.Processes Environmental.Contexts.and.Resources Social.Influences

Emotions Behavioral.Regulation

/STATISTICS=MEAN STDDEV.

**7. Descriptive - At each location, each domain’s mean scores and standard deviation scores**

| **Descriptive Statistics** | | | | |
| --- | --- | --- | --- | --- |
| Location | | N | Mean | Std. Deviation |
| Urban | Knowledge | 428 | 6.46 | 2.82 |
|  | Skills | 428 | 6.36 | 2.69 |
|  | Social.Professional.Role.and.Identity | 428 | 6.93 | 2.76 |
|  | Beliefs.in.Capabilities | 428 | 6.26 | 1.61 |
|  | Optimism | 428 | 5.17 | 1.84 |
|  | Beliefs.in.Consequences | 428 | 6.52 | 2.08 |
|  | Reinforcement | 428 | 5.31 | 2.15 |
|  | Intentions | 428 | 6.14 | 2.90 |
|  | Goals | 428 | 5.80 | 1.68 |
|  | Memory.Attention.and.Decision.Processes | 428 | 6.03 | 1.72 |
|  | Environmental.Contexts.and.Resources | 428 | 6.25 | 2.24 |
|  | Social.Influences | 428 | 6.11 | 2.33 |
|  | Emotions | 428 | 6.30 | 1.79 |
|  | Behavioral.Regulation | 428 | 5.72 | 2.17 |
|  | Valid N (listwise) | 428 |  |  |
| Rural | Knowledge | 121 | 6.89 | 2.87 |
|  | Skills | 121 | 5.74 | 2.06 |
|  | Social.Professional.Role.and.Identity | 121 | 6.65 | 2.64 |
|  | Beliefs.in.Capabilities | 121 | 6.33 | 1.55 |
|  | Optimism | 121 | 5.18 | 1.40 |
|  | Beliefs.in.Consequences | 121 | 6.74 | 1.86 |
|  | Reinforcement | 121 | 4.73 | 1.79 |
|  | Intentions | 121 | 5.91 | 2.94 |
|  | Goals | 121 | 5.86 | 1.53 |
|  | Memory.Attention.and.Decision.Processes | 121 | 5.42 | 1.56 |
|  | Environmental.Contexts.and.Resources | 121 | 6.16 | 1.96 |
|  | Social.Influences | 121 | 6.10 | 1.85 |
|  | Emotions | 121 | 6.16 | 1.40 |
|  | Behavioral.Regulation | 121 | 6.24 | 2.31 |
|  | Valid N (listwise) | 121 |  |  |

SPLIT FILE OFF.

T-TEST GROUPS=ur(1 2)

/MISSING=ANALYSIS

/VARIABLES=Knowledge Skills Social.Professional.Role.and.Identity Beliefs.in.Capabilities

Optimism Beliefs.in.Consequences Reinforcement Intentions Goals

Memory.Attention.and.Decision.Processes Environmental.Contexts.and.Resources Social.Influences

Emotions Behavioral.Regulation

/CRITERIA=CI(.95).

**8. T-Tests - Comparing location domain scores at each domain**

| **Group Statistics** | | | | | |
| --- | --- | --- | --- | --- | --- |
|  | Location | N | Mean | Std. Deviation | Std. Error Mean |
| Knowledge | Urban | 428 | 6.4603 | 2.81715 | .13617 |
|  | Rural | 121 | 6.8884 | 2.87409 | .26128 |
| Skills | Urban | 428 | 6.3575 | 2.68760 | .12991 |
|  | Rural | 121 | 5.7355 | 2.06466 | .18770 |
| Social.Professional.Role.and.Identity | Urban | 428 | 6.9299 | 2.75626 | .13323 |
|  | Rural | 121 | 6.6529 | 2.63853 | .23987 |
| Beliefs.in.Capabilities | Urban | 428 | 6.2648 | 1.61262 | .07795 |
|  | Rural | 121 | 6.3251 | 1.55305 | .14119 |
| Optimism | Urban | 428 | 5.1682 | 1.83665 | .08878 |
|  | Rural | 121 | 5.1777 | 1.39621 | .12693 |
| Beliefs.in.Consequences | Urban | 428 | 6.5187 | 2.08027 | .10055 |
|  | Rural | 121 | 6.7397 | 1.85865 | .16897 |
| Reinforcement | Urban | 428 | 5.3096 | 2.15023 | .10394 |
|  | Rural | 121 | 4.7273 | 1.79002 | .16273 |
| Intentions | Urban | 428 | 6.1379 | 2.89992 | .14017 |
|  | Rural | 121 | 5.9091 | 2.94392 | .26763 |
| Goals | Urban | 428 | 5.7967 | 1.68428 | .08141 |
|  | Rural | 121 | 5.8636 | 1.53433 | .13948 |
| Memory.Attention.and.Decision.Processes | Urban | 428 | 6.0319 | 1.72498 | .08338 |
|  | Rural | 121 | 5.4160 | 1.56127 | .14193 |
| Environmental.Contexts.and.Resources | Urban | 428 | 6.2469 | 2.23742 | .10815 |
|  | Rural | 121 | 6.1570 | 1.95934 | .17812 |
| Social.Influences | Urban | 428 | 6.1051 | 2.32984 | .11262 |
|  | Rural | 121 | 6.0992 | 1.84574 | .16779 |
| Emotions | Urban | 428 | 6.2996 | 1.78647 | .08635 |
|  | Rural | 121 | 6.1591 | 1.40498 | .12773 |
| Behavioral.Regulation | Urban | 428 | 5.7243 | 2.17257 | .10502 |
|  | Rural | 121 | 6.2355 | 2.30947 | .20995 |

| **Independent Samples Test** | | | | | | | | | | | |
| --- | --- | --- | --- | --- | --- | --- | --- | --- | --- | --- | --- |
|  | | Levene's Test for Equality of Variances | | t-test for Equality of Means | | | | | | | |
|  |  | F | Sig. | t | df | Sig. (2-tailed) | Mean Difference | Std. Error Difference | 95% Confidence Interval of the Difference | |  |
|  |  |  |  |  |  |  |  |  | Lower | Upper |  |
| Knowledge | Equal variances assumed | .01 | .94 | -1.47 | 547.00 | .14 | -.43 | .29 | -1.00 | .14 |  |
|  | Equal variances not assumed |  |  | -1.45 | 190.10 | .15 | -.43 | .29 | -1.01 | .15 |  |
| Skills | Equal variances assumed | 24.33 | .00 | 2.36 | 547.00 | .02 | .62 | .26 | .10 | 1.14 |  |
|  | Equal variances not assumed |  |  | 2.72 | 246.60 | .01 | .62 | .23 | .17 | 1.07 |  |
| Social.Professional.Role.and.Identity | Equal variances assumed | .72 | .40 | .99 | 547.00 | .32 | .28 | .28 | -.28 | .83 |  |
|  | Equal variances not assumed |  |  | 1.01 | 200.11 | .31 | .28 | .27 | -.26 | .82 |  |
| Beliefs.in.Capabilities | Equal variances assumed | .02 | .89 | -.37 | 547.00 | .71 | -.06 | .16 | -.38 | .26 |  |
|  | Equal variances not assumed |  |  | -.37 | 199.11 | .71 | -.06 | .16 | -.38 | .26 |  |
| Optimism | Equal variances assumed | 6.41 | .01 | -.05 | 547.00 | .96 | -.01 | .18 | -.36 | .34 |  |
|  | Equal variances not assumed |  |  | -.06 | 249.36 | .95 | -.01 | .15 | -.31 | .30 |  |
| Beliefs.in.Consequences | Equal variances assumed | 2.78 | .10 | -1.06 | 547.00 | .29 | -.22 | .21 | -.63 | .19 |  |
|  | Equal variances not assumed |  |  | -1.12 | 212.55 | .26 | -.22 | .20 | -.61 | .17 |  |
| Reinforcement | Equal variances assumed | 3.41 | .07 | 2.72 | 547.00 | .01 | .58 | .21 | .16 | 1.00 |  |
|  | Equal variances not assumed |  |  | 3.02 | 227.25 | .00 | .58 | .19 | .20 | .96 |  |
| Intentions | Equal variances assumed | .10 | .75 | .76 | 547.00 | .45 | .23 | .30 | -.36 | .82 |  |
|  | Equal variances not assumed |  |  | .76 | 190.83 | .45 | .23 | .30 | -.37 | .82 |  |
| Goals | Equal variances assumed | .30 | .58 | -.39 | 547.00 | .69 | -.07 | .17 | -.40 | .27 |  |
|  | Equal variances not assumed |  |  | -.41 | 208.87 | .68 | -.07 | .16 | -.39 | .25 |  |
| Memory.Attention.and.Decision.Processes | Equal variances assumed | 1.21 | .27 | 3.54 | 547.00 | .00 | .62 | .17 | .27 | .96 |  |
|  | Equal variances not assumed |  |  | 3.74 | 210.09 | .00 | .62 | .16 | .29 | .94 |  |
| Environmental.Contexts.and.Resources | Equal variances assumed | 2.67 | .10 | .40 | 547.00 | .69 | .09 | .22 | -.35 | .53 |  |
|  | Equal variances not assumed |  |  | .43 | 216.52 | .67 | .09 | .21 | -.32 | .50 |  |
| Social.Influences | Equal variances assumed | 8.18 | .00 | .03 | 547.00 | .98 | .01 | .23 | -.45 | .46 |  |
|  | Equal variances not assumed |  |  | .03 | 238.84 | .98 | .01 | .20 | -.39 | .40 |  |
| Emotions | Equal variances assumed | 12.54 | .00 | .80 | 547.00 | .43 | .14 | .18 | -.21 | .49 |  |
|  | Equal variances not assumed |  |  | .91 | 240.64 | .36 | .14 | .15 | -.16 | .44 |  |
| Behavioral.Regulation | Equal variances assumed | 3.21 | .07 | -2.25 | 547.00 | .02 | -.51 | .23 | -.96 | -.07 |  |
|  | Equal variances not assumed |  |  | -2.18 | 184.31 | .03 | -.51 | .23 | -.97 | -.05 |  |

**--------Closed and Opened dataset-----------**

GET

FILE='\\WIMPLE\User54\b\bssmbf\Desktop\Behaviour Insigh Data_EligibleURBAN_For JofHP9.28.sav'.

DATASET NAME DataSet1 WINDOW=FRONT.

GET

FILE='\\WIMPLE\User54\b\bssmbf\Desktop\Behaviour Insigh Data_For JofHP9.28.sav'.

DATASET NAME DataSet2 WINDOW=FRONT.

DATASET ACTIVATE DataSet2.

DATASET CLOSE DataSet1.

USE ALL.

COMPUTE filter_$=(Eligible = 1).

VARIABLE LABELS filter_$ 'Eligible = 1 (FILTER)'.

VALUE LABELS filter_$ 0 'Not Selected' 1 'Selected'.

FORMATS filter_$ (f1.0).

FILTER BY filter_$.

EXECUTE.

SORT CASES BY ur.

SPLIT FILE LAYERED BY ur.

* OMS.

DATASET DECLARE Plum.for.urban.and.rural.

OMS

/SELECT TABLES

/IF COMMANDS=['PLUM'] SUBTYPES=['Parameter Estimates']

/DESTINATION FORMAT=SAV NUMBERED=TableNumber_

OUTFILE='Plum.for.urban.and.rural' VIEWER=YES.

DATASET ACTIVATE DataSet2.

PLUM MMAS_Adherence_Category_For.Ordinal.Regression WITH Knowledge Skills

Social.Professional.Role.and.Identity Beliefs.in.Capabilities Optimism Beliefs.in.Consequences

Reinforcement Intentions Goals Memory.Attention.and.Decision.Processes

Environmental.Contexts.and.Resources Social.Influences Emotions Behavioral.Regulation

/CRITERIA=CIN(95) DELTA(0) LCONVERGE(0) MXITER(100) MXSTEP(5) PCONVERGE(1.0E-6) SINGULAR(1.0E-8)

/LINK=LOGIT

/PRINT=FIT PARAMETER SUMMARY TPARALLEL

/SAVE=ESTPROB PREDCAT PCPROB ACPROB.

**9. PLUM - Ordinal Regression at Urban and Rural Locations**

| **Case Processing Summary** | | | | |
| --- | --- | --- | --- | --- |
| Location | | | N | Marginal Percentage |
| Urban | MMAS_Adherence_Category_For.Ordinal.Regression | Low Adherer | 177 | 41.4% |
|  |  | Medium Adherer | 210 | 49.1% |
|  |  | High Adherer | 41 | 9.6% |
|  | Valid | | 428 | 100.0% |
|  | Missing | | 0 |  |
|  | Total | | 428 |  |
| Rural | MMAS_Adherence_Category_For.Ordinal.Regression | Low Adherer | 43 | 35.5% |
|  |  | Medium Adherer | 70 | 57.9% |
|  |  | High Adherer | 8 | 6.6% |
|  | Valid | | 121 | 100.0% |
|  | Missing | | 0 |  |
|  | Total | | 121 |  |

| **Model Fitting Information** | | | | | |
| --- | --- | --- | --- | --- | --- |
| Location | Model | -2 Log Likelihood | Chi-Square | df | Sig. |
| Urban | Intercept Only | 802.568 |  |  |  |
|  | Final | 745.043 | 57.525 | 14 | .000 |
| Rural | Intercept Only | 209.058 |  |  |  |
|  | Final | 186.304 | 22.753 | 14 | .064 |
| Link function: Logit. | | | | | |

| **Goodness-of-Fit** | | | | |
| --- | --- | --- | --- | --- |
| Location | | Chi-Square | df | Sig. |
| Urban | Pearson | 874.242 | 836 | .174 |
|  | Deviance | 743.657 | 836 | .990 |
| Rural | Pearson | 220.630 | 226 | .588 |
|  | Deviance | 186.304 | 226 | .975 |
| Link function: Logit. | | | | |

| **Pseudo R-Square** | | | | | | | |  |  |  |  |  |  |  |  |  |  |  |
| --- | --- | --- | --- | --- | --- | --- | --- | --- | --- | --- | --- | --- | --- | --- | --- | --- | --- | --- |
| Urban | | Cox and Snell | | | .126 | | |  |  |  |  |  |  |  |  |  |  |  |
|  |  | Nagelkerke | | | .148 | | |  |  |  |  |  |  |  |  |  |  |  |
|  |  | McFadden | | | .072 | | |  |  |  |  |  |  |  |  |  |  |  |
| Rural | | Cox and Snell | | | .171 | | |  |  |  |  |  |  |  |  |  |  |  |
|  |  | Nagelkerke | | | .208 | | |  |  |  |  |  |  |  |  |  |  |  |
|  |  | McFadden | | | .109 | | |  |  |  |  |  |  |  |  |  |  |  |
| Link function: Logit. | | | | | | | |  |  |  |  |  |  |  |  |  |  |  |
| **Parameter Estimates** | | | | | | | | | | | | | | | | | | |
| Location | | | | | | | | Estimate | Std. Error | | Wald | | df | Sig. | | 95% Confidence Interval | |  |
|  |  |  |  |  |  |  |  |  |  |  |  |  |  |  |  | Lower Bound | Upper Bound |  |
| Urban | Threshold | | | [MMAS_Adherence_Category_For.Ordinal.Regression = 1.00] | | | | 3.203 | .740 | | 18.752 | | 1 | .000 | | 1.753 | 4.653 |  |
|  |  |  |  | [MMAS_Adherence_Category_For.Ordinal.Regression = 2.00] | | | | 6.089 | .794 | | 58.813 | | 1 | .000 | | 4.533 | 7.645 |  |
|  | Location | | | Knowledge | | | | .034 | .062 | | .306 | | 1 | .580 | | -.088 | .157 |  |
|  |  |  |  | Skills | | | | .140 | .055 | | 6.430 | | 1 | .011 | | .032 | .248 |  |
|  |  |  |  | Social.Professional.Role.and.Identity | | | | -.071 | .052 | | 1.818 | | 1 | .178 | | -.173 | .032 |  |
|  |  |  |  | Beliefs.in.Capabilities | | | | .090 | .078 | | 1.325 | | 1 | .250 | | -.063 | .243 |  |
|  |  |  |  | Optimism | | | | .069 | .058 | | 1.387 | | 1 | .239 | | -.046 | .183 |  |
|  |  |  |  | Beliefs.in.Consequences | | | | .121 | .066 | | 3.415 | | 1 | .065 | | -.007 | .249 |  |
|  |  |  |  | Reinforcement | | | | .012 | .057 | | .042 | | 1 | .837 | | -.100 | .123 |  |
|  |  |  |  | Intentions | | | | -.103 | .055 | | 3.491 | | 1 | .062 | | -.212 | .005 |  |
|  |  |  |  | Goals | | | | .035 | .064 | | .293 | | 1 | .589 | | -.091 | .161 |  |
|  |  |  |  | Memory.Attention.and.Decision.Processes | | | | .235 | .065 | | 13.279 | | 1 | .000 | | .109 | .362 |  |
|  |  |  |  | Environmental.Contexts.and.Resources | | | | .010 | .074 | | .017 | | 1 | .897 | | -.136 | .155 |  |
|  |  |  |  | Social.Influences | | | | -.143 | .073 | | 3.854 | | 1 | .050 | | -.286 | .000 |  |
|  |  |  |  | Emotions | | | | .111 | .087 | | 1.637 | | 1 | .201 | | -.059 | .280 |  |
|  |  |  |  | Behavioral.Regulation | | | | .060 | .062 | | .918 | | 1 | .338 | | -.062 | .181 |  |
| Rural | Threshold | | | [MMAS_Adherence_Category_For.Ordinal.Regression = 1.00] | | | | 3.930 | 1.667 | | 5.559 | | 1 | .018 | | .663 | 7.197 |  |
|  |  |  |  | [MMAS_Adherence_Category_For.Ordinal.Regression = 2.00] | | | | 7.690 | 1.837 | | 17.527 | | 1 | .000 | | 4.090 | 11.291 |  |
|  | Location | | | Knowledge | | | | -.055 | .176 | | .099 | | 1 | .753 | | -.400 | .289 |  |
|  |  |  |  | Skills | | | | .328 | .138 | | 5.623 | | 1 | .018 | | .057 | .598 |  |
|  |  |  |  | Social.Professional.Role.and.Identity | | | | -.016 | .123 | | .017 | | 1 | .897 | | -.258 | .226 |  |
|  |  |  |  | Beliefs.in.Capabilities | | | | .015 | .195 | | .006 | | 1 | .938 | | -.367 | .398 |  |
|  |  |  |  | Optimism | | | | .043 | .147 | | .084 | | 1 | .773 | | -.246 | .331 |  |
|  |  |  |  | Beliefs.in.Consequences | | | | .157 | .134 | | 1.361 | | 1 | .243 | | -.107 | .421 |  |
|  |  |  |  | Reinforcement | | | | .032 | .130 | | .062 | | 1 | .803 | | -.222 | .286 |  |
|  |  |  |  | Intentions | | | | -.076 | .117 | | .427 | | 1 | .514 | | -.305 | .152 |  |
|  |  |  |  | Goals | | | | -.096 | .159 | | .364 | | 1 | .546 | | -.408 | .216 |  |
|  |  |  |  | Memory.Attention.and.Decision.Processes | | | | .387 | .160 | | 5.842 | | 1 | .016 | | .073 | .700 |  |
|  |  |  |  | Environmental.Contexts.and.Resources | | | | -.003 | .197 | | .000 | | 1 | .987 | | -.389 | .383 |  |
|  |  |  |  | Social.Influences | | | | -.152 | .208 | | .531 | | 1 | .466 | | -.560 | .256 |  |
|  |  |  |  | Emotions | | | | .122 | .236 | | .266 | | 1 | .606 | | -.341 | .585 |  |
|  |  |  |  | Behavioral.Regulation | | | | .138 | .157 | | .772 | | 1 | .380 | | -.170 | .445 |  |
| Link function: Logit. | | | | | | | | | | | | | | | | | | |
| **Test of Parallel Lines^a^** | | | | | | | | | | | | | | |  |  |  |  |
| Location | | | Model | | | -2 Log Likelihood | Chi-Square | | | df | | Sig. | | |  |  |  |  |
| Urban | | | Null Hypothesis | | | 745.043 |  | | |  | |  | | |  |  |  |  |
|  |  |  | General | | | 734.785 | 10.258 | | | 14 | | .743 | | |  |  |  |  |
| Rural | | | Null Hypothesis | | | 186.304 |  | | |  | |  | | |  |  |  |  |
|  |  |  | General | | | 162.950 | 23.354 | | | 14 | | .055 | | |  |  |  |  |
| The null hypothesis states that the location parameters (slope coefficients) are the same across response categories. | | | | | | | | | | | | | | |  |  |  |  |
| a. Link function: Logit. | | | | | | | | | | | | | | |  |  |  |  |

* OMSEND.

OMSEND TAG=['$Id1'].

DATASET ACTIVATE Plum.for.urban.and.rural.

SAVE OUTFILE='\\WIMPLE\User54\b\bssmbf\Desktop\Behaviour Insigh Data_Regression.PLUM_For '+

'JofHP9.28.sav'

/COMPRESSED.

COMPUTE Exp_B = EXP(Estimate).

COMPUTE Lower = EXP(LowerBound).

COMPUTE Upper = EXP(UpperBound).

FORMATS Exp_B Lower Upper (F8.3).

EXECUTE.

DATASET ACTIVATE Plum.for.urban.and.rural.

SAVE OUTFILE='\\WIMPLE\User54\b\bssmbf\Desktop\Behaviour Insigh Data_Regression.PLUM_For '+

'JofHP9.28.sav'

/COMPRESSED.

DATASET ACTIVATE DataSet2.

DATASET CLOSE Plum.for.urban.and.rural.

DATASET ACTIVATE DataSet2.

SAVE OUTFILE='\\WIMPLE\User54\b\bssmbf\Desktop\Behaviour Insigh Data_For JofHP9.28.sav'

/COMPRESSED.

REGRESSION

/MISSING LISTWISE

/STATISTICS COLLIN TOL

/CRITERIA=PIN(.05) POUT(.10)

/NOORIGIN

/DEPENDENT MMAS_Adherence_Category_For.Ordinal.Regression

/METHOD=ENTER Knowledge Skills Social.Professional.Role.and.Identity Beliefs.in.Capabilities

Optimism Beliefs.in.Consequences Reinforcement Intentions Goals

Memory.Attention.and.Decision.Processes Environmental.Contexts.and.Resources Social.Influences

Emotions Behavioral.Regulation.

**10. Regression - Assessing Multicollinarity of Domains at Urban and Rural Locations**

| **Variables Entered/Removed^a^** | | | | | | | |  |
| --- | --- | --- | --- | --- | --- | --- | --- | --- |
| Location | Model | | Variables Entered | Variables Removed | | Method | |  |
| Urban | 1 | | Behavioral.Regulation, Memory.Attention.and.Decision.Processes, Optimism, Goals, Beliefs.in.Capabilities, Reinforcement, Beliefs.in.Consequences, Social.Professional.Role.and.Identity, Skills, Emotions, Intentions, Environmental.Contexts.and.Resources, Social.Influences, Knowledge^b^ | . | | Enter | |  |
| Rural | 1 | | Behavioral.Regulation, Optimism, Reinforcement, Goals, Skills, Beliefs.in.Consequences, Memory.Attention.and.Decision.Processes, Beliefs.in.Capabilities, Social.Professional.Role.and.Identity, Emotions, Intentions, Social.Influences, Environmental.Contexts.and.Resources, Knowledge^b^ | . | | Enter | |  |
| a. Dependent Variable: MMAS_Adherence_Category_For.Ordinal.Regression | | | | | | | |  |
| b. All requested variables entered. | | | | | | | |  |
| **Coefficients^a^** | | | | | | | | |
| Location | Model | | | | Collinearity Statistics | | | |
|  |  |  |  |  | Tolerance | | VIF | |
| Urban | 1 | Knowledge | | | .31 | | 3.23 | |
|  |  | Skills | | | .44 | | 2.28 | |
|  |  | Social.Professional.Role.and.Identity | | | .46 | | 2.20 | |
|  |  | Beliefs.in.Capabilities | | | .61 | | 1.65 | |
|  |  | Optimism | | | .81 | | 1.23 | |
|  |  | Beliefs.in.Consequences | | | .51 | | 1.97 | |
|  |  | Reinforcement | | | .62 | | 1.61 | |
|  |  | Intentions | | | .37 | | 2.68 | |
|  |  | Goals | | | .80 | | 1.25 | |
|  |  | Memory.Attention.and.Decision.Processes | | | .78 | | 1.28 | |
|  |  | Environmental.Contexts.and.Resources | | | .34 | | 2.92 | |
|  |  | Social.Influences | | | .32 | | 3.09 | |
|  |  | Emotions | | | .39 | | 2.55 | |
|  |  | Behavioral.Regulation | | | .51 | | 1.96 | |
| Rural | 1 | Knowledge | | | .14 | | 7.14 | |
|  |  | Skills | | | .47 | | 2.15 | |
|  |  | Social.Professional.Role.and.Identity | | | .33 | | 3.06 | |
|  |  | Beliefs.in.Capabilities | | | .39 | | 2.59 | |
|  |  | Optimism | | | .85 | | 1.17 | |
|  |  | Beliefs.in.Consequences | | | .57 | | 1.75 | |
|  |  | Reinforcement | | | .68 | | 1.47 | |
|  |  | Intentions | | | .30 | | 3.30 | |
|  |  | Goals | | | .60 | | 1.66 | |
|  |  | Memory.Attention.and.Decision.Processes | | | .62 | | 1.62 | |
|  |  | Environmental.Contexts.and.Resources | | | .25 | | 4.02 | |
|  |  | Social.Influences | | | .25 | | 4.03 | |
|  |  | Emotions | | | .33 | | 3.06 | |
|  |  | Behavioral.Regulation | | | .28 | | 3.54 | |
| a. Dependent Variable: MMAS_Adherence_Category_For.Ordinal.Regression | | | | | | | | |

**11. Odds Ratios - A picture of the additional data set generated by the Plum procedure**


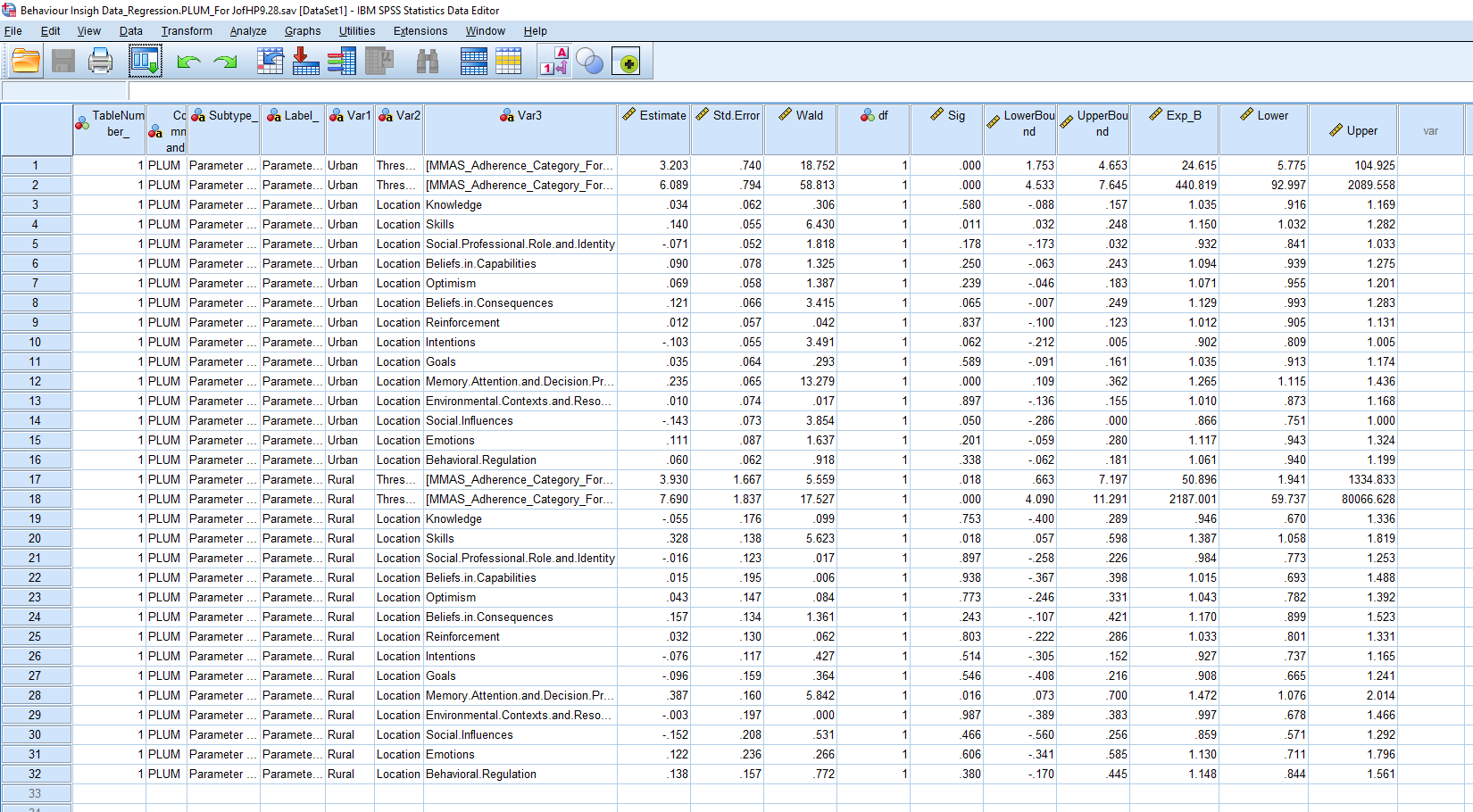


**--------Closed and Opened dataset-----------**

GET

FILE='\\WIMPLE\User54\b\bssmbf\Desktop\Behaviour Insigh Data_For JofHP9.28.sav'.

DATASET NAME DataSet1 WINDOW=FRONT.

FREQUENCIES VARIABLES=ur

/STATISTICS=RANGE MINIMUM MAXIMUM MODE

/ORDER=ANALYSIS.

**12. Frequencies - Total number of participants surveyed at each location**

[DataSet1] \\WIMPLE\User54\b\bssmbf\Desktop\Behaviour Insigh Data_For JofHP9.28.sav

| **Statistics** | | |
| --- | --- | --- |
| Location | | |
| N | Valid | 1892 |
|  | Missing | 0 |
| Mode | | 1 |
| Range | | 1 |
| Minimum | | 1 |
| Maximum | | 2 |

| **Location** | | | | | |
| --- | --- | --- | --- | --- | --- |
|  | | Frequency | Percent | Valid Percent | Cumulative Percent |
| Valid | Urban | 1376 | 72.7 | 72.7 | 72.7 |
|  | Rural | 516 | 27.3 | 27.3 | 100.0 |
|  | Total | 1892 | 100.0 | 100.0 |  |

CROSSTABS

/TABLES=ur BY nq6.Ever.Taken.Antibiotics.YES.NO

/FORMAT=AVALUE TABLES

/STATISTICS=CHISQ PHI

/CELLS=COUNT ROW

/COUNT ROUND CELL.

**13. Crosstabs - Chi square test comparing percentage of participants having taken antibiotics at each location**

| **Case Processing Summary** | | | | | | |
| --- | --- | --- | --- | --- | --- | --- |
|  | Cases | | | | | |
|  | Valid | | Missing | | Total | |
|  | N | Percent | N | Percent | N | Percent |
| Location * Please tell me have you ever taken the antibiotic medication? | 1888 | 99.8% | 4 | 0.2% | 1892 | 100.0% |

| **Location * Please tell me have you ever taken the antibiotic medication? Crosstabulation** | | | | | | | | | | |  |
| --- | --- | --- | --- | --- | --- | --- | --- | --- | --- | --- | --- |
|  | | | | | Please tell me have you ever taken the antibiotic medication? | | | | Total | |  |
|  |  |  |  |  | Yes | | No | |  |  |  |
| Location | Urban | Count | | | 547 | | 825 | | 1372 | |  |
|  |  | % within Location | | | 39.9% | | 60.1% | | 100.0% | |  |
|  | Rural | Count | | | 174 | | 342 | | 516 | |  |
|  |  | % within Location | | | 33.7% | | 66.3% | | 100.0% | |  |
| Total | | Count | | | 721 | | 1167 | | 1888 | |  |
|  |  | % within Location | | | 38.2% | | 61.8% | | 100.0% | |  |
| **Chi-Square Tests** | | | | | | | | | | | |
|  | | | Value | df | | Asymptotic Significance (2-sided) | | Exact Sig. (2-sided) | | Exact Sig. (1-sided) | |
| Pearson Chi-Square | | | 6.004^a^ | 1 | | .014 | |  | |  | |
| Continuity Correction^b^ | | | 5.746 | 1 | | .017 | |  | |  | |
| Likelihood Ratio | | | 6.068 | 1 | | .014 | |  | |  | |
| Fisher's Exact Test | | |  |  | |  | | .015 | | .008 | |
| Linear-by-Linear Association | | | 6.001 | 1 | | .014 | |  | |  | |
| N of Valid Cases | | | 1888 |  | |  | |  | |  | |
| a. 0 cells (0.0%) have expected count less than 5. The minimum expected count is 197.05. | | | | | | | | | | | |
| b. Computed only for a 2x2 table | | | | | | | | | | | |

| **Symmetric Measures** | | | |
| --- | --- | --- | --- |
|  | | Value | Approximate Significance |
| Nominal by Nominal | Phi | .056 | .014 |
|  | Cramer's V | .056 | .014 |
| N of Valid Cases | | 1888 |  |

CROSSTABS

/TABLES=ur BY Eligible

/FORMAT=AVALUE TABLES

/STATISTICS=CHISQ PHI

/CELLS=COUNT ROW

/COUNT ROUND CELL.

**13. Crosstabs - Chi square test comparing percentage of participants who completed all survey items (i.e. eligible) at each location**

| **Case Processing Summary** | | | | | | |
| --- | --- | --- | --- | --- | --- | --- |
|  | Cases | | | | | |
|  | Valid | | Missing | | Total | |
|  | N | Percent | N | Percent | N | Percent |
| Location * Eligible | 721 | 38.1% | 1171 | 61.9% | 1892 | 100.0% |

| **Location * Eligible Crosstabulation** | | | | | |
| --- | --- | --- | --- | --- | --- |
|  | | | Eligible | | Total |
|  |  |  | No-Not Eligible for analyses | Yes-Eligible for analyses |  |
| Location | Urban | Count | 119 | 428 | 547 |
|  |  | % within Location | 21.8% | 78.2% | 100.0% |
|  | Rural | Count | 53 | 121 | 174 |
|  |  | % within Location | 30.5% | 69.5% | 100.0% |
| Total | | Count | 172 | 549 | 721 |
|  |  | % within Location | 23.9% | 76.1% | 100.0% |

| **Chi-Square Tests** | | | | | |
| --- | --- | --- | --- | --- | --- |
|  | Value | df | Asymptotic Significance (2-sided) | Exact Sig. (2-sided) | Exact Sig. (1-sided) |
| Pearson Chi-Square | 5.507^a^ | 1 | .019 |  |  |
| Continuity Correction^b^ | 5.038 | 1 | .025 |  |  |
| Likelihood Ratio | 5.306 | 1 | .021 |  |  |
| Fisher's Exact Test |  |  |  | .024 | .013 |
| Linear-by-Linear Association | 5.499 | 1 | .019 |  |  |
| N of Valid Cases | 721 |  |  |  |  |
| a. 0 cells (0.0%) have expected count less than 5. The minimum expected count is 41.51. | | | | | |
| b. Computed only for a 2x2 table | | | | | |

| **Symmetric Measures** | | | |
| --- | --- | --- | --- |
|  | | Value | Approximate Significance |
| Nominal by Nominal | Phi | -.087 | .019 |
|  | Cramer's V | .087 | .019 |
| N of Valid Cases | | 721 |  |

USE ALL.

COMPUTE filter_$=(Eligible = 1).

VARIABLE LABELS filter_$ 'Eligible = 1 (FILTER)'.

VALUE LABELS filter_$ 0 'Not Selected' 1 'Selected'.

FORMATS filter_$ (f1.0).

FILTER BY filter_$.

EXECUTE.

CROSSTABS

/TABLES=ur BY sex

/FORMAT=AVALUE TABLES

/STATISTICS=CHISQ PHI

/CELLS=COUNT ROW

/COUNT ROUND CELL.

**15. Crosstabs - Chi square test comparing percentage of eligible participants who identified as female at each location**

| **Case Processing Summary** | | | | | | |
| --- | --- | --- | --- | --- | --- | --- |
|  | Cases | | | | | |
|  | Valid | | Missing | | Total | |
|  | N | Percent | N | Percent | N | Percent |
| Location * Gender | 549 | 100.0% | 0 | 0.0% | 549 | 100.0% |

| **Location * Gender Crosstabulation** | | | | | |
| --- | --- | --- | --- | --- | --- |
|  | | | Gender | | Total |
|  |  |  | Male | Female |  |
| Location | Urban | Count | 187 | 241 | 428 |
|  |  | % within Location | 43.7% | 56.3% | 100.0% |
|  | Rural | Count | 81 | 40 | 121 |
|  |  | % within Location | 66.9% | 33.1% | 100.0% |
| Total | | Count | 268 | 281 | 549 |
|  |  | % within Location | 48.8% | 51.2% | 100.0% |

| **Chi-Square Tests** | | | | | |
| --- | --- | --- | --- | --- | --- |
|  | Value | df | Asymptotic Significance (2-sided) | Exact Sig. (2-sided) | Exact Sig. (1-sided) |
| Pearson Chi-Square | 20.409^a^ | 1 | .000 |  |  |
| Continuity Correction^b^ | 19.489 | 1 | .000 |  |  |
| Likelihood Ratio | 20.695 | 1 | .000 |  |  |
| Fisher's Exact Test |  |  |  | .000 | .000 |
| Linear-by-Linear Association | 20.372 | 1 | .000 |  |  |
| N of Valid Cases | 549 |  |  |  |  |
| a. 0 cells (0.0%) have expected count less than 5. The minimum expected count is 59.07. | | | | | |
| b. Computed only for a 2x2 table | | | | | |

| **Symmetric Measures** | | | |
| --- | --- | --- | --- |
|  | | Value | Approximate Significance |
| Nominal by Nominal | Phi | -.193 | .000 |
|  | Cramer's V | .193 | .000 |
| N of Valid Cases | | 549 |  |

CROSSTABS

/TABLES=ur BY age

/FORMAT=AVALUE TABLES

/STATISTICS=CHISQ PHI

/CELLS=COUNT ROW

/COUNT ROUND CELL.

**16. Crosstabs - Chi square test comparing percentage of eligible participants across age groups each location**

| **Case Processing Summary** | | | | | | |
| --- | --- | --- | --- | --- | --- | --- |
|  | Cases | | | | | |
|  | Valid | | Missing | | Total | |
|  | N | Percent | N | Percent | N | Percent |
| Location * Age of the Respondent | 549 | 100.0% | 0 | 0.0% | 549 | 100.0% |

| **Location * Age of the Respondent Crosstabulation** | | | | | | | | | | |
| --- | --- | --- | --- | --- | --- | --- | --- | --- | --- | --- |
|  | | | | | Age of the Respondent | | | | | Total |
|  |  |  |  |  | Under 30 | | 30 – 50 | 50+ | |  |
| Location | Urban | Count | | | 165 | | 229 | 34 | | 428 |
|  |  | % within Location | | | 38.6% | | 53.5% | 7.9% | | 100.0% |
|  | Rural | Count | | | 43 | | 70 | 8 | | 121 |
|  |  | % within Location | | | 35.5% | | 57.9% | 6.6% | | 100.0% |
| Total | | Count | | | 208 | | 299 | 42 | | 549 |
|  |  | % within Location | | | 37.9% | | 54.5% | 7.7% | | 100.0% |
| **Chi-Square Tests** | | | | | | | | |  |  |
|  | | | Value | df | | Asymptotic Significance (2-sided) | | |  |  |
| Pearson Chi-Square | | | .772^a^ | 2 | | .680 | | |  |  |
| Likelihood Ratio | | | .779 | 2 | | .677 | | |  |  |
| Linear-by-Linear Association | | | .073 | 1 | | .787 | | |  |  |
| N of Valid Cases | | | 549 |  | |  | | |  |  |
| a. 0 cells (0.0%) have expected count less than 5. The minimum expected count is 9.26. | | | | | | | | |  |  |

| **Symmetric Measures** | | | |
| --- | --- | --- | --- |
|  | | Value | Approximate Significance |
| Nominal by Nominal | Phi | .038 | .680 |
|  | Cramer's V | .038 | .680 |
| N of Valid Cases | | 549 |  |

CROSSTABS

/TABLES=ur BY MMAS_Adherence_Category

/FORMAT=AVALUE TABLES

/STATISTICS=CHISQ PHI

/CELLS=COUNT ROW

/COUNT ROUND CELL.

**17. Crosstabs - Chi square test comparing percentage of eligible participants across adherence categories at each location**

| **Case Processing Summary** | | | | | | |
| --- | --- | --- | --- | --- | --- | --- |
|  | Cases | | | | | |
|  | Valid | | Missing | | Total | |
|  | N | Percent | N | Percent | N | Percent |
| Location * MMAS_Adherence_Category | 549 | 100.0% | 0 | 0.0% | 549 | 100.0% |

| **Location * MMAS_Adherence_Category Crosstabulation** | | | | | | |
| --- | --- | --- | --- | --- | --- | --- |
|  | | | MMAS_Adherence_Category | | | Total |
|  |  |  | High Adherer | Medium Adherer | Low Adherer |  |
| Location | Urban | Count | 41 | 210 | 177 | 428 |
|  |  | % within Location | 9.6% | 49.1% | 41.4% | 100.0% |
|  | Rural | Count | 8 | 70 | 43 | 121 |
|  |  | % within Location | 6.6% | 57.9% | 35.5% | 100.0% |
| Total | | Count | 49 | 280 | 220 | 549 |
|  |  | % within Location | 8.9% | 51.0% | 40.1% | 100.0% |

| **Chi-Square Tests** | | | |
| --- | --- | --- | --- |
|  | Value | df | Asymptotic Significance (2-sided) |
| Pearson Chi-Square | 3.155^a^ | 2 | .206 |
| Likelihood Ratio | 3.203 | 2 | .202 |
| Linear-by-Linear Association | .195 | 1 | .659 |
| N of Valid Cases | 549 |  |  |
| a. 0 cells (0.0%) have expected count less than 5. The minimum expected count is 10.80. | | | |

| **Symmetric Measures** | | | |
| --- | --- | --- | --- |
|  | | Value | Approximate Significance |
| Nominal by Nominal | Phi | .076 | .206 |
|  | Cramer's V | .076 | .206 |
| N of Valid Cases | | 549 |  |

CROSSTABS

/TABLES=ur BY inc_n

/FORMAT=AVALUE TABLES

/STATISTICS=CHISQ PHI

/CELLS=COUNT ROW

/COUNT ROUND CELL.

**18. Crosstabs - Chi square test comparing percentage of eligible participants across income categories at each location**

| **Case Processing Summary** | | | | | | |
| --- | --- | --- | --- | --- | --- | --- |
|  | Cases | | | | | |
|  | Valid | | Missing | | Total | |
|  | N | Percent | N | Percent | N | Percent |
| Location * Monthly HHIncome | 483 | 88.0% | 66 | 12.0% | 549 | 100.0% |

| **Chi-Square Tests** | | | |
| --- | --- | --- | --- |
|  | Value | df | Asymptotic Significance (2-sided) |
| Pearson Chi-Square | 39.244^a^ | 3 | .000 |
| Likelihood Ratio | 36.008 | 3 | .000 |
| Linear-by-Linear Association | 26.458 | 1 | .000 |
| N of Valid Cases | 483 |  |  |
| a. 0 cells (0.0%) have expected count less than 5. The minimum expected count is 13.53. | | | |

| **Symmetric Measures** | | | |
| --- | --- | --- | --- |
|  | | Value | Approximate Significance |
| Nominal by Nominal | Phi | .285 | .000 |
|  | Cramer's V | .285 | .000 |
| N of Valid Cases | | 483 |  |
