# Supplementary material for: Choice of methods can determine which behavioral determinates are identified for targeting in future behavior change interventions: Increasing antibiotic adherence in Pakistan
Source: J Health Psychol. 2020 Oct 4;27(4):1006–13. doi: 10.1177/1359105320962267 (PMC8855384; doi:10.1177/1359105320962267)
Supplement: Syntax_Analysis_Guide_31-July-2020 – for Choice of methods can determine which behavioral determinates are identified for targeting in future behavior change interventions: Increasing antibiotic adherence in Pakistan [file Syntax_Analysis_Guide_31-July-2020.docx]

**Syntax for Main Analyses**

**(SPSS version 26)**

**Rank Order Domain Scores Syntax**

DESCRIPTIVES VARIABLES=Knowledge Skills Social.Professional.Role.and.Identity

Beliefs.in.Capabilities Optimism Beliefs.in.Consequences Reinforcement Intentions Goals

Memory.Attention.and.Decision.Processes Environmental.Contexts.and.Resources Social.Influences

Emotions Behavioral.Regulation

/STATISTICS=MEAN STDDEV.

**T-test Syntax**

DATASET ACTIVATE DataSet1.

T-TEST GROUPS=ur(1 2)

/MISSING=ANALYSIS

/VARIABLES=Knowledge Skills Social.Professional.Role.and.Identity Beliefs.in.Capabilities

Optimism Beliefs.in.Consequences Reinforcement Intentions Goals

Memory.Attention.and.Decision.Processes Environmental.Contexts.and.Resources Social.Influences

Emotions Behavioral.Regulation

/CRITERIA=CI(.95).

**Ordinal Regression Syntax**

**Syntax 1. On: Behaviour Insigh Data_For JofHP9.28**

PLUM MMAS_Adherence_Category_For.Ordinal.Regression WITH Knowledge Skills

Social.Professional.Role.and.Identity Beliefs.in.Capabilities Optimism Beliefs.in.Consequences

Reinforcement Intentions Goals Memory.Attention.and.Decision.Processes

Environmental.Contexts.and.Resources Social.Influences Emotions Behavioral.Regulation

/CRITERIA=CIN(95) DELTA(0) LCONVERGE(0) MXITER(100) MXSTEP(5) PCONVERGE(1.0E-6) SINGULAR(1.0E-8)

/LINK=LOGIT

/PRINT=FIT PARAMETER SUMMARY TPARALLEL

/SAVE=ESTPROB PREDCAT PCPROB ACPROB.

**Syntax 2. Behaviour Insigh Data_Regression.PLUM_For JofHP9.28**

COMPUTE Exp_B = EXP(Estimate).

COMPUTE Lower = EXP(LowerBound).

COMPUTE Upper = EXP(UpperBound).

FORMATS Exp_B Lower Upper (F8.3).

EXECUTE.
