# Supplementary material for: Choice of methods can determine which behavioral determinates are identified for targeting in future behavior change interventions: Increasing antibiotic adherence in Pakistan
Source: J Health Psychol. 2020 Oct 4;27(4):1006–13. doi: 10.1177/1359105320962267 (PMC8855384; doi:10.1177/1359105320962267)
Supplement: Supplementary_Materials – Supplemental material for Choice of methods can determine which behavioral determinates are identified for targeting in future behavior change interventions: Increasing antibiotic adherence in Pakistan [file Supplementary_Materials.pdf]

Supplementary Materials 1.

Translations of Items from English to Roman Urdu. RS indicates a reverse scored item.

Scale		English		Roman Urdu
Medication Adherence	Q1	Do you ever forget to take your antibiotic medication?	Q1	Kya aap kabhi apni antibiotic dawai khaani bhoolay hain?
	Q2	Are you careless at times about taking your antibiotic medication?	Q2	Kya aap ne kabhi apni antibiotic dawai khaanay mein laparwahi ki hai?
	Q3	Sometimes, if you feel worse when you take the antibiotics, do you stop taking it?	Q3	Kabhi kabaar agar antibiotic dawai khanay se aap ziada baddtar mehsoos kerein, tu kya aap dawai khaani chor daitay hain?
	Q4	When you feel better, do you sometimes stop taking the antibiotics?	Q4	Agar aap mehsoos karein keh aap theek ho gae hain, tu kya aap antibiotic dawai khaani chor daitay hian?
Knowledge **(An awareness of the existence of something)***	D1.1	I know that antibiotic medication course should be finished	D1.1	mujhay maaloon hai keh antibiotic course poora mukammal karna chahiye
	D1.2	I know how to complete the antibiotic medication course	D1.2	mujhay antibiotic course mukammal karna aata hai
Skills **(An ability or proficiency acquired through practice)**	D2.1	I have the skills to complete the antibiotic medication course	D2.1	mein antibiotic course mukammal karnay ki maharat rakhta hoon
Social/professional role and identity **(A coherent set of behaviors and displayed personal qualities of an individual in a social or work setting)**	D3.1	It is my responsibility as a patient to complete the antibiotic medication course	D3.1	yeh meray mareez honay ki zimmedari hai keh mein antibiotic course mukammal karoon
Beliefs about capabilities **(Acceptance of the truth, reality, or validity about an ability, talent, or facility that a person can put to constructive use)**	D4.1	I am confident that I can complete the antibiotic medication course even if I am not motivated	D4.1	mujhay yaqeen hai keh agar mera dil na bhi chahay tu mein antibiotic course mukammal kar sakta hoon
	D4.2	I am confident that if I wanted I could complete the antibiotic medication course	D4.2	mujhay yaqeen hai keh agar mein chahoon tu mein antibiotic course mukammal kar sakta hoon
	D4.3	For me, it is difficult to complete the antibiotic medication course (RS)	D4.3	meray liye antibiotic course mukammal karna mushkil hai
Optimism	D5.1	With regards to completing the antibiotic medication course, nothing bad will happen	D5.1	antibiotic course mukammal karnay se kuch bura nahin hota

**(The confidence that things will happen for the best or that desired goals will be attained)**	D5.2	With regards to completing the antibiotic medication course, if something can go wrong it will (RS)	D5.2	agar antibiotic course mukammal karnay se kuch bura ho sakta hai tu who zaroor ho ga
Beliefs about own consequences **(Acceptance of the truth, reality, or validity about outcomes of a behavior in a given situation)**	D6.1	For me, completing the antibiotic medication course is useless (RS)	D6.1	antibiotic course mukammal karna bekaar hai
	D6.2	If I complete the antibiotic medication course. It will benefit me	D6.2	agar mein antibiotic course mukammal karoon, tu iss mein mera faida hai
Reinforcement **(Increasing the probability of a response by arranging a dependent relationship, or contingency, between the response and a given stimulus)**	D7.1	Whenever I complete the antibiotic medication course, I feel recognition from people who are important to me	D7.1	jab bhi mein antibiotic course mukammal karta hoon tu mujhay unn logon se jo meray lye eham hain hosla afzai milti hai
	D7.2	Whenever I complete the antibiotic medication course, I get rewarded	D7.2	jab bhi mein antibiotic course mukammal karta hoon tu mujhay inaam milta hai
Intentions **(A conscious decision to perform a behavior or a resolve to act in a certain way)**	D8.1	I intend to complete the antibiotic medication course next time	D8.1	agli dafa mein antibiotic course mukammal karnay ka irada kerta hoon
Goals **(Mental representations of outcomes or end states that an individual wants to achieve)**	D9.1	I have a clear plan of how I will complete the antibiotic medication course	D9.1	meray paas antibiotic course mukammal karnay ka waazeh mansooba hai
	D9.2	For me covering something else on my agenda is often a higher priority than completing the antibiotic medication course (RS)	D9.2	aksar meray liye baaki kaam niptana antibiotic course khatam karnay se ziada zaroori hotay hain
Memory attention and decision processes **(The ability to retain information, focus selectively on aspects of the environment and choose between two or more alternatives)**	D10.1	For me completing the antibiotic medication course is easy to remember	D10.1	mujhay anitbiotic course mukmmal karna aasani se yaad rehta hai
	D10.2	I often forget to complete the antibiotic medication course (RS)	D10.2	mein aksar antibiotic course mukammal karna bhool jaata hoon
	D10.3	I get distracted from completing the antibiotic medication course (RS)	D10.3	aksar meri tawajjuh antibiotic course mukammal karnay se hat jaati hai
Environmental context and resources **(Any circumstance of a person's situation or environment that discourages or encourages the development of skills and abilities, independence, social competence, and adaptive behavior)**	D11.1	In my society, completing the antibiotic medication course is common	D11.1	meray ilaaqay mein antibiotic course mukammal karna aam hai
	D11.2	Within the socio-political context there is good communication between myself and my doctor	D11.2	siyaasi o samaaji lihaz se meray aur meray doctor ke darmiyaan acha raabta hai
	D11.3	Prior to giving the prescription, doctor advised me to complete the antibiotic medication course	D11.3	dawai ki parchi denay se pehlay, doctor ne mujhay antibiotic course mukammal karnay ki hadayat di thi

Social influences<br>**(Those interpersonal processes that can cause individuals to change their thoughts, feelings, or behaviors)**	D12.1	My friends and family are helpful in my completion of the antibiotic medication course	D12.1	meray dost aur ghar walay antibiotic course mukammal karnay mein meri madad kertay hain
	D12.2	Most people who are important to me think that I should complete the antibiotic medication course	D12.2	ziada tar log jo meray liye eham hain samajhtay hain keh mujhay antibiotic course mukammal karna chahiye
	D12.3	People I know complete their antibiotic medication course	D12.3	mein jin bhi logon ko jaanta hoon, who apna antibiotic course mukammal kartay hain
Emotions<br>**(A complex reaction pattern, involving experiential, behavioral, and physiological elements, by which the individual attempts to deal with a personally significant matter or event)**	D13.1	I generally feel worried or concerned about not completing the antibiotic medication course (RS)	D13.1	mein apna antibiotic course mukammal na karnay ke baray mein pareshan rehta hoon
	D13.2	I generally feel good about completing the antibiotic medication course	D13.2	antibiotic course mukammal kar ke mein khushi mehsoos karta hoon
	D13.3	I generally enjoy my normal day to day activities	D13.3	mein apni roz marra ki zindagi enjoy karta hoon
	D13.4	I generally feel unhappy and depressed (RS)	D13.4	aam taur pe mein nakhoosh aur udaas rehta hoon
Behavioral regulation<br>**(Anything aimed at managing or changing objectively observed or measured actions)**	D14.1	I usually complete my antibiotic medication course without thinking	D14.1	mein ziada sochay baghair antibiotic course mukammal ker leta hoon
	D14.2	I keep track of my progress in completing the antibiotic medication course	D14.2	antibiotic course mukammal karnay ke liye mein apni peshraft ko dekhta rehta hoon

* The definitions of each domain come from Table 2 of Cane, et al. (2012), which are based on definitions from the American Psychological Associations’ Dictionary of Psychology (Vandenbos, 2007).

Supplementary Materials 2.

Descriptive statistics for all participants and then urban and rural participants for demographics and survey items.

		Location	
		urban	rural
Surveyed by Gallup		1,376	516
Indicated taking antibiotics [a]		547	174
Completed all Adherence and TDF items [b]		428	121
Gender [c]	Female	241 (56.3%)	40 (33.1%)
Age [d]	under 30 years	165 (38.6%)	43 (35.5%)
	30 to 50 years	229 (53.5%)	70 (35.5%)
	over 50	34 (7.9%)	8 (6.6%)
Monthly Income [e]	no response	44 (10.0%)	22 (18.2%)
	up to 7,000	35 (8.2%)	31 (25.6%)
	7,001 to 15,000	128 (29.9%)	25 (28.9%)
	15001 to 30,000	141 (32.9%)	26 (21.5%)
	more than 30,000	80 (18.7%)	7 (5.8%)
Adherence Classification [d]	low	177 (41.4%)	43 (35.5%)
	medium	210 (49.1%)	70 (57.9%)
	high	41 (9.6%)	8 (6.6%)
Domain Scores	Optimism	5.17 (1.84)	5.18 (1.40)
	Reinforcement	5.31 (2.15)	4.73 (1.79)
	Goals	5.80 (1.68)	5.86 (1.53)

Behavioral regulation	5.72 (2.17)	6.24 (2.31)
Memory attention …	6.03 (1.72)	5.42 (1.56)
Intentions	6.14 (2.90)	5.91 (2.94)
Social Influences	6.11 (2.33)	6.10 (1.85)
Skills	6.36 (2.69)	5.74 (2.06)
Environmental …	6.25 (2.24)	6.16 (1.96)
Emotions	6.30 (1.79)	6.16 (1.40)
Beliefs about capabilities	6.26 (1.61)	6.33 (1.55)
Knowledge	6.46 (2.82)	6.89 (2.87)
Beliefs about conseq…	6.52 (2.08)	6.74 (1.86)
Social/professional role…	6.93 (2.76)	6.65 (2.64)

[a] The percentage of participants indicating having taken antibiotics was higher in the in urban than rural groups ($\chi 2(1) = 6.00$, $p = .01$, $\varphi = .06$).

[b] The percentage of participants completing all the survey items was higher in urban than rural locations ($\chi 2(1) = 5.51$, $p = .02$, $\varphi = .09$).

[c] There were more females in the urban group than the rural group ($\chi 2(1) = 20.41$, $p < .001$, $\varphi = .19$).

[d] Neither participants' age ($\chi 2(2) = 0.77$, $p = .68$ $\varphi = .04$) nor adherence classification ($\chi 2(2) = 3.16$, $p = .21$) differed across locations.

[e] Participants in the urban group had higher incomes than the rural group ($\chi 2(3) = 39.24$, $p < .001$, $\varphi = .29$); Note that only 483 participants stated their income.

Supplementary Materials 3.

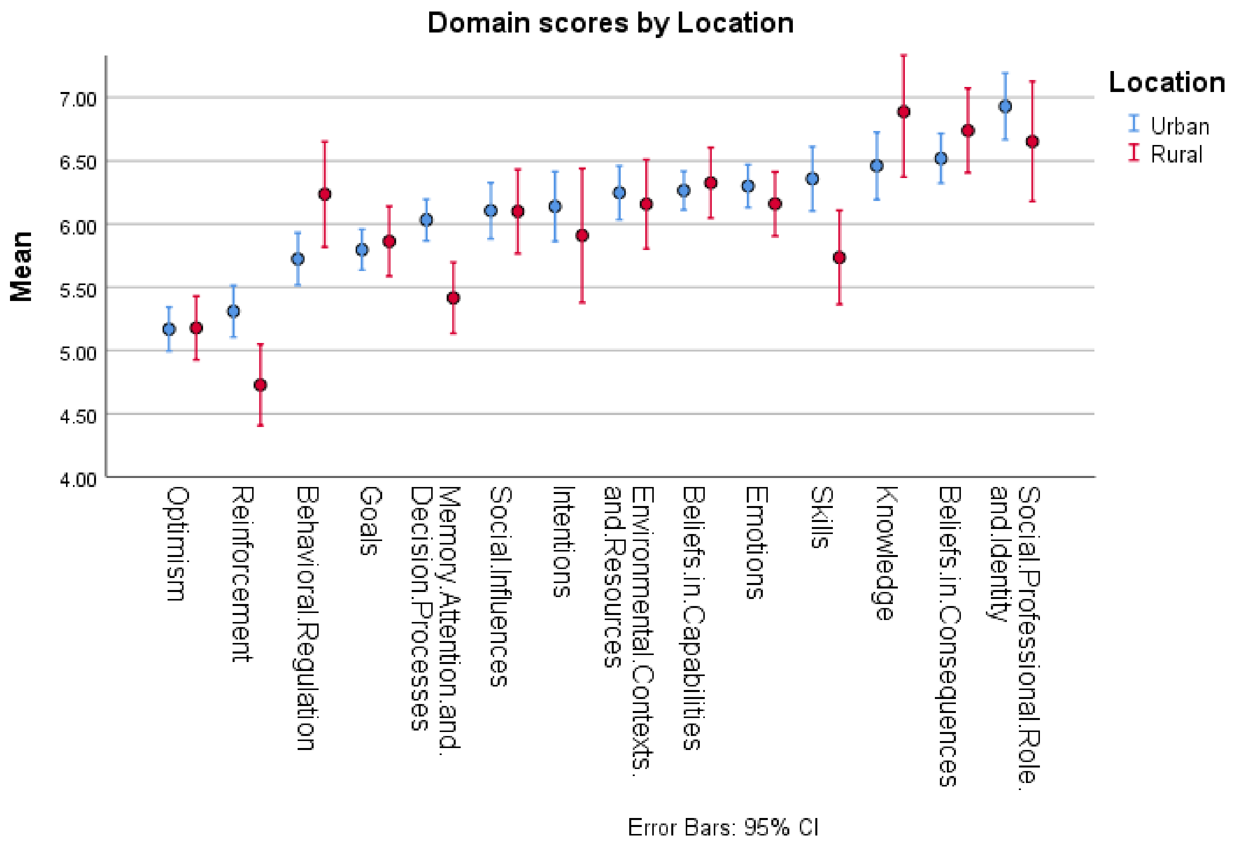

Independent samples T-test analyses comparing urban and rural participants at each domain.

Domain	Equal Variance Assumed	Levene's Test for Equality of Variances		t-test for Equality of Means				
		F	Sig.	t	df	Sig. (2-tailed)	Mean Difference	Std. Error Difference
Optimism	No	6.41	0.01	-0.06	249.36	0.95	-0.01	0.15
Reinforcement	Yes	3.41	0.07	2.72	547.00	0.01	0.58	0.21
Goals	Yes	0.30	0.58	-0.39	547.00	0.69	-0.07	0.17
Behavioral regulation	Yes	3.21	0.07	-2.25	547.00	0.02	-0.51	0.23
Memory attention …	Yes	1.21	0.27	3.54	547.00	0.00	0.62	0.17
Intentions	Yes	0.10	0.75	0.76	547.00	0.45	0.23	0.30
Social Influences	No	8.18	0.00	0.03	238.84	0.98	0.01	0.20
Skills	No	24.33	0.00	2.72	246.60	0.01	0.62	0.23
Environmental …	Yes	2.67	0.10	0.40	547.00	0.69	0.09	0.22
Emotions	No	12.54	0.00	0.91	240.64	0.36	0.14	0.15
Beliefs about capabilities	No	0.02	0.89	-0.37	199.11	0.71	-0.06	0.16
Knowledge	No	0.01	0.94	-1.45	190.10	0.15	-0.43	0.29
Beliefs about conseq…	Yes	2.78	0.10	-1.06	547.00	0.29	-0.22	0.21
Social/professional role…	Yes	0.72	0.40	0.99	547.00	0.32	0.28	0.28

Supplementary Materials 4.

Ordinal regression analysis results for urban and rural participants separately.

	Urban						Rural					
	Wald	df	Sig	Odds Ratio	95% CI		Wald	df	Sig	Odds Ratio	95% CI	
Domains					Lower	Upper					Lower	Upper
Knowledge	0.31	1.00	0.58	1.04	0.92	1.17	0.10	1.00	0.75	0.95	0.67	1.34
Skills	6.43	1.00	0.01*	1.15	1.03	1.28	5.62	1.00	0.02*	1.39	1.06	1.82
Social/professional role …	1.82	1.00	0.18	0.93	0.84	1.03	0.02	1.00	0.90	0.98	0.77	1.25
Beliefs about capabilities	1.33	1.00	0.25	1.09	0.94	1.28	0.01	1.00	0.94	1.02	0.69	1.49
Optimism	1.39	1.00	0.24	1.07	0.96	1.20	0.08	1.00	0.77	1.04	0.78	1.39
Beliefs about consequences	3.42	1.00	0.07	1.13	0.99	1.28	1.36	1.00	0.24	1.17	0.90	1.52
Reinforcement	0.04	1.00	0.84	1.01	0.91	1.13	0.06	1.00	0.80	1.03	0.80	1.33
Intentions	3.49	1.00	0.06	0.90	0.81	1.01	0.43	1.00	0.51	0.93	0.74	1.17
Goals	0.29	1.00	0.59	1.04	0.91	1.17	0.36	1.00	0.55	0.91	0.67	1.24
Memory attention …	13.28	1.00	0.00**	1.27	1.12	1.44	5.84	1.00	0.02*	1.47	1.08	2.01
Environmental context …	0.02	1.00	0.90	1.01	0.87	1.17	0.00	1.00	0.99	1.00	0.68	1.47
Social influences	3.85	1.00	0.05	0.87	0.75	1.00	0.53	1.00	0.47	0.86	0.57	1.29
Emotions	1.64	1.00	0.20	1.12	0.94	1.32	0.27	1.00	0.61	1.13	0.71	1.80
Behavioral regulation	0.92	1.00	0.34	1.06	0.94	1.20	0.77	1.00	0.38	1.15	0.84	1.56

* indicates $p < 0.05$, ** indicates $p < 0.01$
